# Enhanced vertical railway track quality index with dynamic responses from moving trains

**DOI:** 10.1016/j.heliyon.2024.e38670

**Published:** 2024-09-30

**Authors:** Siwarak Unsiwilai, Wassamon Phusakulkajorn, Chen Shen, Arjen Zoeteman, Rolf Dollevoet, Alfredo Núñez, Zili Li

**Affiliations:** a Section of Railway Engineering, Faculty of Civil Engineering and Geosciences, Delft University of Technology, Delft, 2628CN, the Netherlands; b ProRail, Utrecht, 3511EP, the Netherlands

**Keywords:** Axle box acceleration, Condition monitoring, Feature fusion, Track quality index

## Abstract

The conventional vertical track quality index (TQI) based on the standard deviation of longitudinal levels yields standardized railway track condition assessment. Nevertheless, its capability to identify problems is limited, particularly in the ballast and substructure layers when abrupt changes affect train-track interaction. Previous research shows that dynamic responses from moving trains via axle box acceleration (ABA) measurements can quantify abrupt changes in the vertical dynamic responses. Thus, this paper proposes a framework to design an enhanced vertical TQI, called EnVTQI, by integrating track longitudinal levels and dynamic responses from ABA measurements. First, measured ABA signals are processed to mitigate the influence of variation in measurement speed. Then, substructure and ballast-related features are extracted, including scale average wavelet power (SAWP) in the ranges 0.04 m^-1^ to 0.33 m^-1^ (substructure) and 1.25 m^-1^ to 2.50 m^-1^ (ballast). This enables identifying track conditions at different track layers. Finally, EnVTQI is determined by weight averaging between the conventional vertical TQI and the ABA features from moving trains. The performance of EnVTQI is evaluated based on 48 segments of a 200-m track on a Dutch railway line. The results indicate that EnVTQI helps to distinguish track segments that cause poor train-track interaction, which the conventional TQI does not indicate. EnVTQI can supplement the conventional TQI, improving the effectiveness of track maintenance decision-making.

## 1 Introduction

**Fig. 1:**
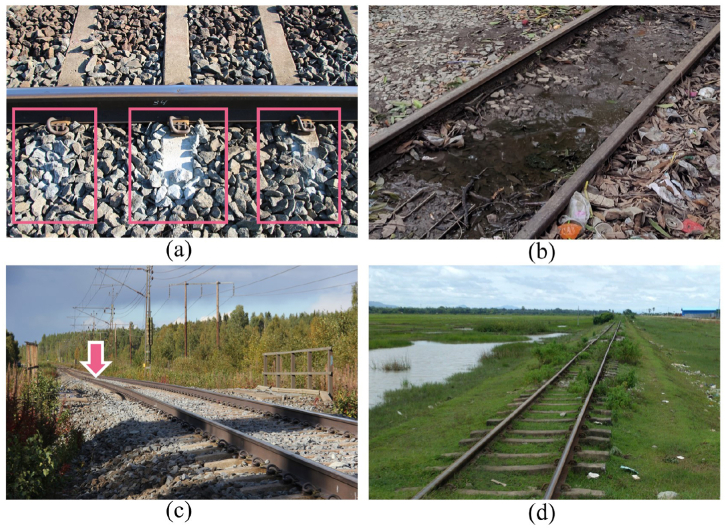
Degraded track segments: (a) ballast breakage, (b) mud pumping and excessive undrained water, (c) excessive track settlement as indicated by the arrow, and (d) vegetation in the track.

The ballasted tracks are utilized globally due to their numerous advantages, such as low construction costs, less complexity in design and construction processes, and simplicity of maintenance [1]. The ballast layer and several underneath engineering materials layers, known as the substructure, play an essential role in supporting the track superstructure, i.e., sleepers and rails. The main functions of the substructure are transferring traffic loads, facilitating drainage, and maintaining track alignments [2]. The degradation of ballast and substructure layers leads to poor track quality, resulting in broken ballast, excessive mud, and track settlement. In addition, vegetation and excessive undrained water on the track surface suggest a high moisture content in the substructure layer, resulting in a lower bearing capacity of materials [3], as shown in Fig. 1(a–d). These degraded track segments can cause abrupt changes in train-track interaction, which increases risks and safety concerns and reduces the service quality of train operations.

Track geometry measurements using track recording vehicles [4] are the standard practice in the railway industry for track inspection at the network level. To assess the overall quality of railway tracks, different track quality indices (TQI) have been developed based on statistical analyses of track geometry parameters and combined by using mathematical methods. TQIs are then used by railway authorities or infrastructure managers to standardize track quality and define maintenance requirements. Examples of conventional TQIs are the combined standard deviation (CoSD) of the European standard [5], the Q index of the Swedish national railway [6], the Chinese TQI [7], the US track roughness index [8], and the FRA track geometry index [9]. In Ref. [10], the accuracy, sensitivity, data required, and specificity of TQIs, including CoSD as the baseline, are evaluated based on synthetic track geometry data. As expected, the findings show that different TQIs perform differently in indicating track segments that need maintenance. When selecting a TQI, their tradeoffs are to be considered. For example, the US track roughness index and FRA Track Geometry Index (TGI) show high sensitivity to changes in geometry parameters, which might be easily biased by noise or error in measurement. In Ref. [11], the characteristics of 14 TQIs have been studied based on measurement data. The results show that the standard deviation-based TQIs perform well in assessing overall track condition but might be biased due to track gauge widening in the curved track segments.

Some studies have focused on data-driven and machine-learning methods to extract information from track geometry parameters as an alternative approach. Using data analytics, those applications can provide track geometry forecasts [12] and track geometry degradation predictions [13]. In Ref. [14], principal components analysis (PCA) is used for dimension reduction of track geometry parameters. The results show that more than 90 % of the variance can be explained by the first three principal components (PCs). In addition, the PCs can be considered directly as TQIs since they perform better in defective segment identification than conventional TQIs. In Ref. [15], the dimension reduction of track geometry parameters using T-stochastic neighbor embedding (T-SNE), a nonlinear method, is compared with PCA, a linear method. While the two methods can represent track quality, T-SNE tends to overfit the training and test datasets, resulting in false defect prediction. In Ref. [16], a stochastic TQI is developed to deal with uncertainty in measurements of track geometry parameters, in which the Bayesian analysis is its core. In Ref. [17], the relationship between track geometry parameters and the lateral to vertical wheel load ratio has been studied using neural network models. The relationships obtained are then used as part of the track geometry inspection technology. Nevertheless, further understanding of the physical meaning of the results from data-driven and machine learning approaches is required by including the physical interpretability of the results.

Track quality assessment based on track geometry parameters has some limitations. For instance, track geometry measurements cannot always capture abrupt changes in train-track interaction [17,18]. Moreover, measurement frequency at a particular location might be limited due to the availability of track recording vehicles. Thus, measurement techniques implemented using in-service trains are becoming the focus of the railway industry. This approach can minimize the abovementioned concerns due to the advantages of the instrumented in-service trains operating daily. Thus, vehicle responses can be obtained frequently as inputs for track quality assessment. However, the challenge is extracting useful information from measured data and standardizing such a type of measurement. According to the literature, several studies have been conducted to find solutions to use vehicle responses for assessing track condition. In Ref. [19], linear regression models are developed to estimate the longitudinal level from bogie vertical acceleration signals. Then, the root-mean-square values of the estimated longitudinal level can be used as a track condition indicator. In Ref. [20], a method to estimate track vertical and lateral irregularities using bogies and axle box acceleration signals is developed. Several layers of filtering methods, including Kalman filter, bandpass filters, and amplitude and phase compensation filters, are applied to analyze acceleration signals to obtain estimated track irregularities. In Ref. [21], a Kalman filter-based model is developed to estimate lateral track irregularity using input from a gyroscope and accelerometers at a wheelset and a bogie frame. Besides estimating track irregularities, some studies develop a track condition indicator derived from measured vehicle responses. In Ref. [22], the continuous wavelet transform is conducted on the simulated vertical vehicle body acceleration from the 2-DOF model while the train passes various track conditions. Then, the summation of wavelet coefficients of the acceleration signal can be considered an indicator to identify damage locations. In Ref. [23], vertical vehicle body acceleration is the input. Then, the developed algorithm is applied to simplify the input into a bump pattern, whose characteristics, such as magnitude, can be considered an indicator. In Ref. [24], a support vector machine classifier with a linear kernel is used to determine the most robust features from car body acceleration in detecting track changes. Those outcomes from vehicle responses can be used separately or supplementary to conventional TQIs for more effective track maintenance planning rather than relying solely on track geometry information from dedicated track recording vehicles.

Track condition assessment based solely on track geometry parameters provides some constraints, while previous research works have demonstrated the potential of utilizing vehicle responses for track condition assessment. Hence, those aspects motivated us to enhance the performance in track condition assessment by integrating track geometry parameters and vehicle responses. This paper proposes a framework to design an enhanced vertical track quality index, called EnVTQI, by combining track geometry and vehicle responses in the vertical direction. Track quality regarding the vertical direction is considered since deviation in the longitudinal level, which is relevant to track vertical irregularities, has been reported to be highly relevant to overall track quality [25]. We focus on the axle box acceleration (ABA) measurement regarding vehicle responses. Measuring ABA is cost-efficient and not complex when implemented on most types of railway vehicles, including passenger trains that operate daily. Several studies have been conducted on ABA measurements for rail defects and irregularities detection [[26], [27], [28], [29], [30], [31]], but a limited number of studies are related to track quality assessment. In Ref. [32], a method to measure track vertical stiffness, a parameter representing a relationship between applied load and overall track deformation, through ABA signals is developed. In Ref. [33], a combined approach between physic and data-driven models is developed to evaluate stiffness at different layers, i.e., railpad and ballast, at the same time.

In this paper, the main contributions are the following.

1) A framework for designing an enhanced vertical track quality index (EnVTQI) by fusing longitudinal level with features from the vertical ABA signals.
2) A method for reducing the influence of measurement speed variation to allow the use of vertical ABA signals that are measured under the operational condition of commercial trains.
3) Validation of the framework on the estimation of ballast and substructure layer conditions.

The remaining sections of this paper are organized as follows. Section 2 describes measurements at railway tracks, which are sources of input for designing EnVTQI. Section 3 explains a proposed framework for designing EnVTQI of a particular track segment. Section 4 presents results and discussions on the performance evaluation of EnVTQI. Finally, conclusions and suggestions for further work are presented in Section 5.

## 2 Inputs required for EnVTQI

### 2.1 Track information

**Fig. 2:**
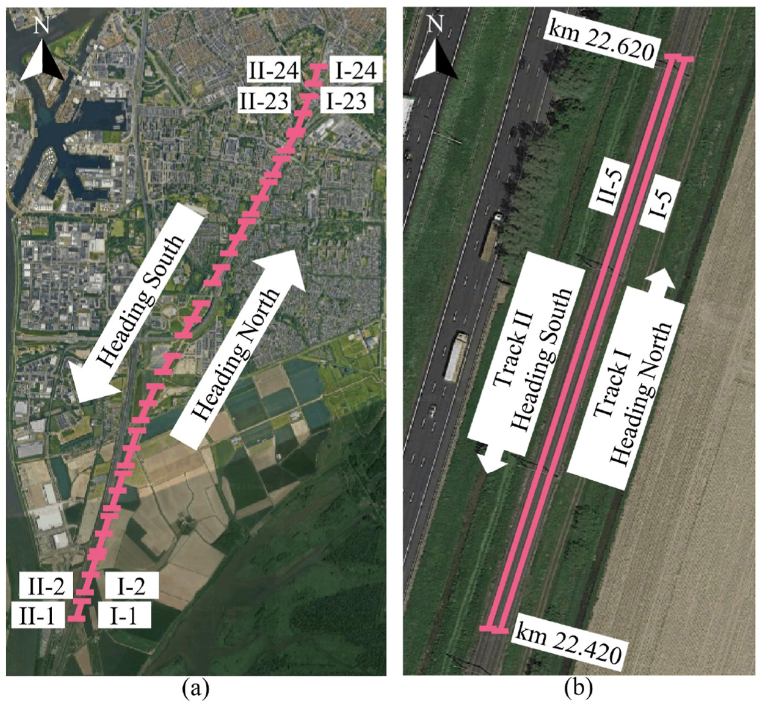
Case study track segments: (a) location of 48 segments, (b) zoom-in detail of track segments I-5 and II-5 (source of satellite photos: Google Maps).

In this study, we analyze track segments located on a Dutch railway line, which consists of 2 tracks that are dedicated to a fixed travel direction. Track-I is for operational traffic heading to the North, and Track-II is for the opposite direction. The study considers mostly straight segments. Then, segments are defined with criteria that each segment is 200 m long, following EN 13848-6 [5], and consists of no civil structures, such as bridges or level crossings, which lead to varying ballast and substructure condition. In addition, some track segments are also excluded due to the composition of track components, such as insulated rail joints, switches, and crossings, resulting in isolated discontinuities of train-track interaction. In addition, the Dutch railway infrastructure has specific criteria for condition assessment of those mentioned civil structures and track components [34]. These criteria align with the finding from Ref. [25] that track segments with different characteristics, such as curvature, embankment thickness, and inclusion with civil structures or joints, show local deterioration rates. Thus, we excluded civil structures and track components from the further analysis, allowing a comparison between track segments with similar structural characteristics. According to the mentioned criteria, 48 of the 200-m-long conventional track segments from both tracks, namely I-1 to I-24 for Track-I and II-1 to II-24 for Track-II, will be evaluated for the case study, as shown in Fig. 2(a and b).

### 2.2 Track longitudinal level measurement

**Fig. 3:**
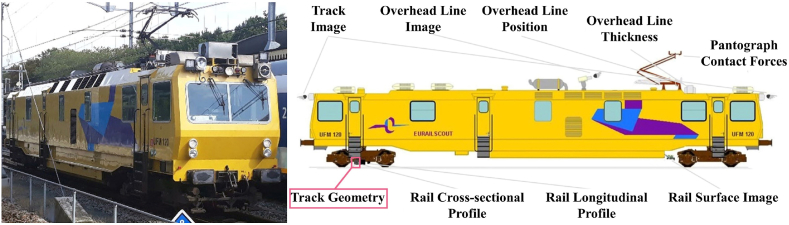
Track recording vehicle, in which the geometry measurement module is shown in the box (source of photos: ProRail).

ProRail, the infrastructure manager for the Dutch railway network, signs performance-based contracts for track maintenance activities with the outsource contractors. As a part of the contracts, contractors must conduct track geometry measurements with track recording vehicles, as shown in Fig. 3, at least once a year. Then, the information from measurements is centralized at the ProRail railway infrastructure monitoring database, called the Branche Breed Monitoring Systeem (BBMS, in Dutch). All datasets in the BBMS system, including track geometry datasets, are reported as corresponding to the reference track kilometer position.

Among measured track geometry parameters, this study considers using only longitudinal levels directly obtained from BBMS. Two obtained longitudinal levels correspond to rail *r* ∈ {L, R}, in which L is the left rail, and R is the right rail. Those signals are already processed with a bandpass filter within a wavelength range between 3 m and 25 m, and the signals have been reported in the space domain with a resolution of 0.25 m, according to EN 13848-1 [35]. Hence, the longitudinal level *LL* of rail *r* at the track location *x* can be defined as *LL*_*r*_(*x*). For a 200 m track segment, *LL*_*r*_ consists of 801 data points per rail. In this paper, one dataset of longitudinal levels measured in 2019 is obtained from BBMS for further analysis on designing EnVTQI.

### 2.3 Axle box acceleration (ABA) measurement

#### 2.3.1 ABA measurement system

**Fig. 4:**
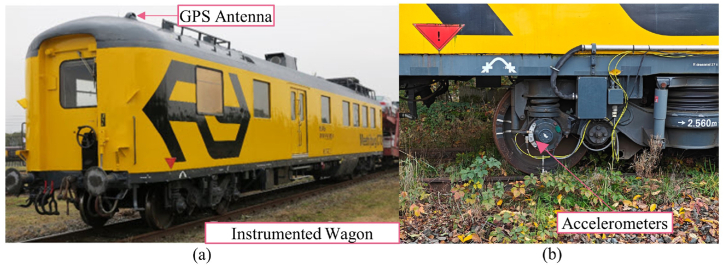
The ABA measurement system instrumented on the dedicated wagon: (a) a GPS antenna (source of photo: TU Delft OpenCourseWare), (b) accelerometers attached to an axle box.

The ABA measurement system, as shown in Fig. 4(a and b), developed by the Section of Railway Engineering, TU Delft [36], consists of three major components. The first component is a set of acceleration sensors, a one-directional accelerometer is attached to a particular axle box of the wagon. This study considers only signals from accelerometers in the vertical direction. The second component is speed and positioning sensors, a tachometer and a global positioning system (GPS) unit with real-time kinematic positioning function to determine the measurement speed and corresponding track position of the wagon. The third component is a data acquisition system (DAQ) unit with in-house developed control software for recording and synchronizing multiple signals from all available sensors. Since the ABA measurement system is designed for a wide range of applications in railway infrastructure condition monitoring (including shortwave defect irregularities detection), a sampling rate of 25.6 kHz is used in the current configuration. ABA systems are currently being implemented in various types of railway vehicles in different countries, including daily passenger trains. In this study, the data collected comes from an instrumented wagon from TU Delft dedicated to railway research purposes. In this setup, loading can be considered almost constant in the different measurements, while an important source of variability will come from speed variations, which are analyzed in this paper.

Based on the configuration of the measurement system, eight acceleration signals are considered from four wheelsets *w* ∈ {w1, w2, w3, w4}, each wheelset with two wheels that correspond to rails *r* ∈ {L, R}, the left (L) and right (R) rails. The acceleration *a* at time instant *t* for wheelset *w* and rail *r* is defined in the time domain as *a*_*w,r*_(*t*). The location of the wheelset *w* at time instant *t* is *x*_*w*_(*t*). Hence, after synchronization with the registered reference information in the BBMS system, such as GPS coordinates of reference kilometers, the acceleration signal can be evaluated as a function of the track location *x* or in the space domain as *a*_*w,r*_(*x*), where location *x* corresponds to the BBMS reference track kilometer positioning.

The number of *a*_*w,r*_ data points per a 200 m track segment depends on the measurement speed. For instance, it could be up to 2.56 × 10^5^ data points from one axle box when an average measurement speed is 20 m/s. In May 2019, two rounds of ABA measurement campaigns were conducted on the case study track segments. These two datasets are used for further analysis in the following sections.

#### 2.3.2 Scale average wavelet power of ABA signals

ABA signals have varied responses due to changes in speed, axle load, and track condition at particular locations. In this paper, we used ABA signals from the dedicated instrumented wagon. Thus, it can be considered that the axle load of the instrumented wagon does not change significantly in different measurements. Therefore, the variations in ABA signals are mainly caused by speed and track conditions. Instead of directly utilizing ABA signals in the space domain, this study analyzes ABA signals in the space-spatial frequency domain, with the ABA signals being synchronized in the time domain with their corresponding position. The signals in the space-spatial frequency domain are converted from signals in the time-frequency domain. The spatial frequency corresponds to the inverse of the wavelength, calculated by dividing the measured frequency by the measured speed, obtained as in Ref. [31].

Since ABA signals are non-stationary signals, wavelet analysis is one of the well-known analysis methods. In this paper, ABA signals in the space domain are transformed into the space-spatial domain using the continuous wavelet transform (CWT) [37]. The wavelet power spectrum (WPS) of ABA signals, a matrix of energy at a specific location (with respect to time synchronization) and frequency (relative to scale), is a product of CWT. Finally, the scale average wavelet power (SAWP) of ABA signals is calculated to investigate the variation of WPS within a considered frequency range along the segment. SAWP can be defined as follows:

Eq. 1 $$SAWP_{w,r}\left(x\right)=\frac{\delta_{j}\delta_{t}}{C_{\delta }}\sum_{j=j1}^{j2}\frac{{\left|\sum_{n^{\prime }=0}^{N-1}a_{w,r}\left(n^{\prime }\right)\psi^{*}\left(\frac{\left(n^{\prime }-n\right)\delta_{t}}{s_{j}}\right)\right|}^{2}}{s_{j}}$$

where *SAWP*_*w,r*_(*x*) is the SAWP of ABA signal at wheel *w* and rail *r*, at location *x* and within the wavelet scale *s* from *s*_*j*1_ to *s*_*j*2_, *δ*_*j*_ is the scale step, *δ*_*t*_ is the time interval between data points, *C*_*δ*_ is the empirically derived constant of the wavelet function*, N* is the number of data points in a considered space frame, *n′* = 0, …, *N*-1, *a*_*w,r*_*(n′)* is the ABA signal at an instant location *x* = *n′*, *n* is the variable for the continuous translation, and *Ψ* is the wavelet mother function, in which the Morlet is selected in the study. The function *Ψ ∗* is a family of wavelets derived from the mother wavelet by translations and scaling, and ∗ refers to the complex conjugate.

According to the literature and findings from our previous studies, the vertical condition of the substructure layer is related to vertical track irregularities in the wavelength range of 3–25 m [31,38,39]. In addition, findings from the measurement campaign in Ref. [40] show that the variation of ballast layer properties exhibits a relationship with SAWP of ABA signals in the wavelength range of sleeper interval. Hence, in this study, we considered SAWP from two spatial frequencies (inverse of the wavelength). Firstly, *SAWP*_S_ corresponds to the spatial frequency range of 0.04 m^-1^ to 0.33 m^-1^ or to irregularities in the wavelength from 3 m to 25 m, which is related to the condition of the substructure layer. Secondly, *SAWP*_B_ corresponds to the spatial frequency range of 1.25 m^-1^ to 2.50 m^-1^ or corresponding to irregularities in the wavelength from 0.4 m to 0.8 m, which covers the 0.6 m of a nominal distance between sleepers in the Dutch railway lines, including ±0.2 m of uncertainty bandwidth. In this range, SAWP is related to the condition of the ballast layer.

## 3 The EnVTQI design

**Fig. 5:**
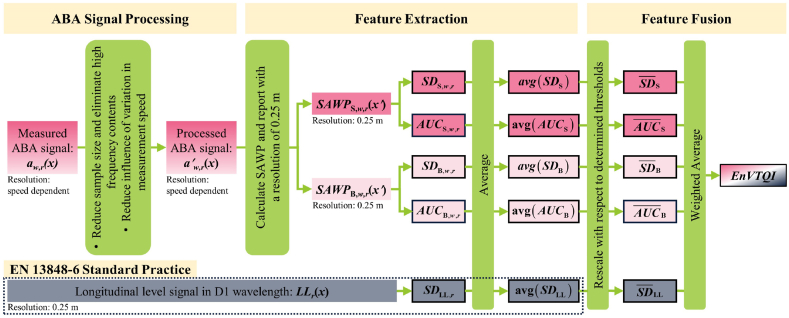
A framework for designing EnVTQI of a particular track segment.

This section presents a framework for designing an EnVTQI of a considered track segment, a 200 m distance each for this study. The framework consists of three main steps: 1) ABA signal processing, 2) Feature extraction from SAWP of ABA signals and longitudinal levels, and 3) Feature fusion method for determining EnVTQI, as shown in Fig. 5. A detailed description of each step can be found in the following subsections.

### 3.1 ABA signals processing

SAWP from two spatial frequency ranges, 3 m–25 m, corresponding to the substructure layer, and 0.4 m–0.8 m, corresponding to the ballast layer, are used for designing EnVTQI. However, calculating SAWP directly from measured ABA signals might consume unnecessary calculation efforts since the signals still contain irrelevant information, such as high-frequency contents corresponding to shortwave irregularities. Further, ABA signals are speed-dependent, so a method is needed to handle the consequences of the speed effect. Hence, processing steps on measured ABA signals should be conducted and are discussed in this section.

#### 3.1.1 Reduce sample size and eliminate high-frequency contents

According to the configuration of our ABA measurement system, ABA signals were recorded at the fixed sampling rate of 25.6 kHz to guarantee that the small defects on the rail surface were captured. According to the literature, responses of ballast and substructure layers are much lower in frequency [[41], [42], [43]]. Thus, a lower sampling rate can be considered for practical applications of ABA systems that analyze ballast and substructure layer-related problems. The fixed sampling rate makes the spatial resolution of ABA signals dependent on measurement speed. A higher speed of the instrumented wagon provides a lower spatial resolution. For example, for a typical train operational speed on the Dutch railway lines of 140 km/h (38.9 m/s), the lowest spatial resolution of ABA signals is 1.52 × 10^−3^ m. This resolution is much higher than the longitudinal levels, whose spatial resolution is 0.25 m. The relationship between passing frequency (*f*), wavelength (*λ*), and passing speed (*v*) is:

Eq. 2 $$f=\frac{v}{\lambda }$$

The shortest considered wavelength for this study is 0.4 m. Regarding a typical train operational speed at 140 km/h (38.9 m/s), the highest corresponding frequency to be considered is 97.2 Hz. In this study, most measurements are below 100 km/h. The desired sampling rate and cutoff frequency must correspond to the operational speed. Thus, we consider a resampling from 25.6 kHz to 256 Hz by applying the standard MATLAB resample function [44] to reduce the sample size and a low pass filtering at 100 Hz. The processed signals are then used for the next steps.

#### 3.1.2 Reduce influence from variation in measurement speed

ABA responses are dependent on running speeds and changes in axle load. In this paper, ABA signals are from our dedicated instrumented wagon, where the axle load can be considered nearly constant during different measurements. Therefore, we consider the effect of measurement speed to be an important source of variability. SAWP from different measurement speeds shows a similar pattern at a particular location [31]. However, the magnitude of SAWP is higher when the measurement speed is higher. Therefore, this step considers reducing the influence of variation in measurement speeds on the SAWP, making an EnVTQI evaluation independent of the measurement speed. We propose an approach to reduce the influence of speed as follows:

Eq. 3 $$a_{w,r}^{\prime }\left(x\right)=\frac{a_{w,r}\left(x\right)}{v^{2}}$$

where *a*′_*w*,*r*_(*x*) is the processed ABA signal from wheel *w* and rail *r* at location *x*, after reducing speed influence, *a*_*w*,*r*_(*x*) is the processed ABA from the previous step, and *v*^2^ is the square of the average measurement speed when the instrumented wagon passes by the considered track segment.

**Fig. 6:**
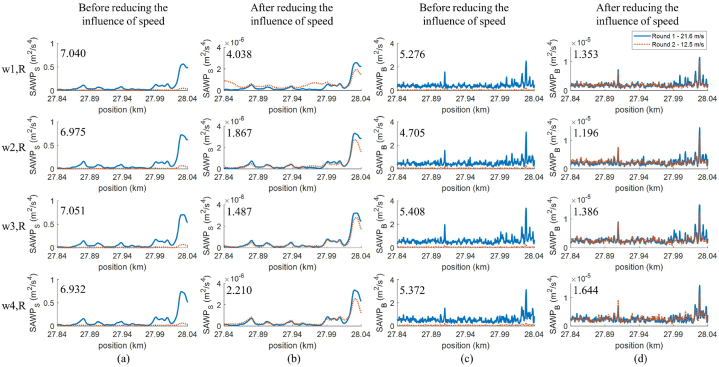
*SAWP*_S_ and *SAWP*_B_ of ABA signals at the right rail of the segment II-24: (a) and (c) before reducing the influence of speed, (b) and (d) after reducing the influence of speed. The top-left number is the scaled Euclidean distance between 2 corresponding signals.

The ABA signals at the right rail of segment II-24 are considered as an example to evaluate the performance of the proposed approach. Two measurements with average speeds of 21.6 m/s and 12.5 m/s were conducted on this segment on the same day of May 2019. Therefore, the condition of the track can be considered identical between the two measurements. SAWP from ABA signals before and after reducing the influence of speeds are shown in Fig. 6(a–d). It can be noticed that the similarity, especially magnitude, of *SAWP*_S_ and *SAWP*_B_ between the two measurements increases after reducing the influence of speed.

The Euclidean distance is proposed as an indicator to assess the similarity between 2 signals, as follows.

Eq. 4 $$d\left(p,q\right)=\sqrt{\sum_{i=1}^{n}{\left(q_{i}-p_{i}\right)}^{2}}$$

where *d*(p,q) is the Euclidean distance between signals p and q, *p*_*i*_ and *q*_*i*_ are the values of signal p and q at index *i*, and n is the signals p and q length. In this case, we consider SAWP for 200 m at the spatial resolution of 0.25 m, resulting in a similar signal length at 801 samples despite the difference in measurement speeds.

**Fig. 7:**
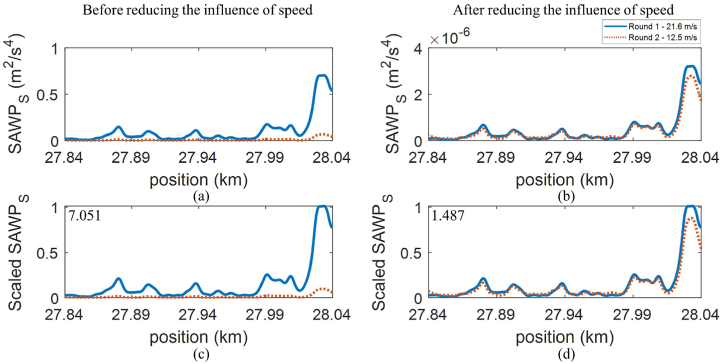
*SAWP*_S_ of ABA signal from wheel 3, the right, at the segment II-24: (a) and (c) are before reducing the influence of speed, (b) and (d) after reducing the influence of speed, (c) and (d) scaled SAWP where the top-left number is corresponding to the scaled Euclidean distance.

The lower distance between 2 signals suggests a higher similarity of those signals, while the zero distance indicates ideally similar signals. Please note that the magnitude of SAWP signals before and after reducing the influence of speed are different. Thus, scaling on SAWP should be conducted before calculating the Euclidean distance to ensure a fair comparison. Fig. 7(a–d) show the example of SAWP_S,w3,R_ before and after reducing the influence of speed and their corresponding scaled SAWP. The value in the top-left of the scaled SAWP plots (Fig. 7(c and d)) corresponds to the scaled Euclidean distance. It can be indicated that the proposed method can reduce the influence of speed since the distance is reduced from 7.051 to 1.487, increasing the similarity between signals from two rounds of measurement. The scaled Euclidean distance between the signals of the remaining wheels can be found in Fig. 6(a–d).

Regarding visual observation and the scaled Euclidean distance, it can be concluded that even though the influence of speed variation still occurs, it is significantly reduced by this proposed approach.

### 3.2 Feature extraction from input signals

In this step, we consider longitudinal levels and SAWPs of ABA signals as input signals. Then, the most relevant features are extracted from input signals to determine EnVTQI. The steps are discussed next.

#### 3.2.1 Feature from longitudinal levels

According to EN 13848-6 [5], the standard practice for quantifying the vertical track quality is based on the standard deviation of longitudinal levels. First, the quality of the measured longitudinal level datasets should be considered. Environmental factors, different measurement conditions, malfunctioning sensors, and other uncontrolled uncertainties might affect the measurement results, such as noise interference and position shifting [45]. Then, preprocessing, such as position alignment or removal of invalid measured values, is required to increase the accuracy of the standard deviation. Then, the standard deviation of the longitudinal level at the left rail, *SD*_LL*,*L_, and the right rail, *SD*_LL*,*R_, can be separately calculated as follows:

Eq. 5 $$SD_{k}=\sqrt{\frac{\sum_{i=1}^{N}{\left(k\left(x_{i}\right)-\bar{k}\right)}^{2}}{N-1}}$$

where *SD*_*k*_ is the standard deviation of signal *k*, *k*(*x*_*i*_) is the value of signal *k* at position *x*_*i*_, $\bar{k}$ is the average value of signal *k*, and *N* is the data size of signal *k*. Then, *SD*_LL*,*L_ and *SD*_LL*,*R_ are averaged into a single value, *avg*(*SD*_LL_), and it is considered as a representative feature from the longitudinal levels of a particular track segment.

#### 3.2.2 SAWP calculation

After reducing the influence of speed variation, the processed ABA signals, *a*′_*w*,*r*_(*x*), are inputs for SAWP calculation, according to Eq. (1). Then, *SAWP*_S,*w,r*_, which corresponds to the condition of the substructure layer, and *SAWP*_B,*w,r*_, which corresponds to the condition of the ballast layer, are obtained from eight ABA signals measured at eight corresponding axle boxes at wheelset *w* and rail *r*. Finally, the SAWP related to *x*′ (same position as reported by the longitudinal level) is obtained with a 0.25 m spatial resolution as follows:

Eq. 6 $$SAWP_{w,r}\left(x^{\prime }\right)=\frac{\sum_{n\in Ν}SAWP_{w,r}\left(x\left(n\right)\right)}{\left|Ν\right|},Ν=\left\{n,\left|x\left(n\right)-x^{\prime }\right|\leq 0.125\;m\right\}$$

where *SAWP*_*w,r*_(*x′*) is the SAWP of the ABA signal from wheel *w* and rail *r* at location *x*′, in which *x*′ is the same position as reported by longitudinal level signals at an interval of 0.25 m. The number of data points |Ν| depends on the measurement speed. By selecting a similar spatial resolution between SAWP and the longitudinal level, we aim to produce a fusion of features that allows us to determine EnVTQI. In addition, a spatial resolution at 0.25 m is approximately half of the sleeper interval that is appropriate for detecting changes or defects in the level of an individual sleeper.

#### 3.2.3 Features from SAWP of ABA signals

*SAWP*_S_ and *SAWP*_B_ are derived products of ABA signals related to substructure and ballast layers, respectively. For designing an enhanced track quality index for a particular track segment in which ABA signals are incorporated, the relevant features to track quality are extracted from SAWP. In this study, two types of handcraft features are considered as follows.

First, a variation of SAWP is considered. Good-quality track segments should provide less variation in track parameters. For example, according to EN 13848-6 [5], track segments that provide lower *avg*(*SD*_LL_) are considered in better quality classes than segments with higher *avg*(*SD*_LL_). Following the mentioned criteria, the standard deviation of SAWP is considered to be one of the handcraft features, which can be calculated using Eq. (6). Then, *SD*_S,*w,r*_, the standard deviation of *SAWP*_S,*w,r*_, and *SD*_B,*w,r*_, the standard deviation of *SAWP*_B,*w,r*_ corresponding to eight axle boxes at wheelset *w* rail *r,* are obtained.

Second, ABA energy is considered to be another handcraft feature. It has been reported that higher ABA energy can be found where severe conditions track components corresponding to various wavelength irregularities are located [31,46,47]. This criterion can be considered the principal characteristic of ABA signals. Thus, ABA energy corresponding to a particular track segment is considered and can be quantified by an area under the SAWP curve (AUC) as follows:

Eq. 7 $$AUC_{k}=\sum_{i=1}^{N}\frac{\left(k\left(x_{i}\right)+k\left(x_{i+1}\right)\right)\Delta x}{2}$$

where *AUC*_*k*_ is the area under the curve of signal *k*, *k*(*x*_*i*_) and *k*(*x*_*i*+1_) are the value of signal *k* at position *x*_*i*_ and the right after position *x*_*i*+1_, Δ*x* is the distance between position *x*_*i*_ and *x*_*i*+1_.

**Fig. 8:**
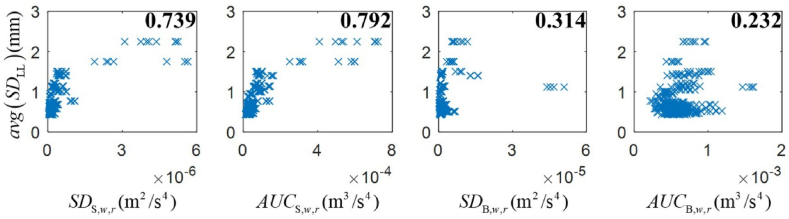
Correlation plots between the average standard deviation of longitudinal levels and features from *SAWP*_S_ and *SAWP*_B_. The top-right number is the Pearson correlation between the paired features of each subplot.

After Eq. (7), *AUC*_S*,w,r*_*,* the area under the *SAWP*_S,*w,r*_ curve, and *AUC*_B*,w,r*_, the area under the *SAWP*_B*,w,r*_ corresponding to eight axle boxes at wheelset *w* rail *r,* are obtained. In total, the ABA signal from a particular axle box at wheelset *w* and rail *r* provides four handcraft features: *SD*_S,*w,r*_, *AUC*_S,*w,r*_, *SD*_B,*w,r*_, and *AUC*_B,*w,r*_. Fig. 8 illustrates the correlations between *avg*(*SD*_LL_) of the 48 case study track segments and handcraft features corresponding to ABA signals from the eight axle boxes during measurement round 1 in 2019. All handcraft features show a positive Pearson correlation to *avg*(*SD*_LL_), with a high correlation of 0.792 and 0.739 for *AUC*_S,*w,r*_ and *SD*_S,*w,r*_. From this analysis, we observe measurements that are outliers and features that are not highly correlated. These locations are interesting in this work, as they are characterized by ABA responses that deviate from *avg*(*SD*_LL_), suggesting local phenomena not captured by track geometry.

### 3.3 Feature fusion

EnVTQI is determined by fusing features from longitudinal levels and ABA signals. EnVTQI is designed to be a single value representing the quality of the track for ease of implementation. According to EN 13848-6, *avg*(*SD*_LL_) is calculated from longitudinal levels corresponding to two rails to represent a vertical TQI of a particular track segment. This principle is adopted in determining EnVTQI, as described next.

**Fig. 9:**
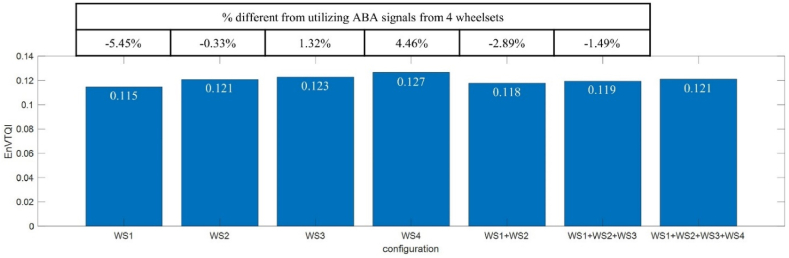
EnVTQI of segment II-24 regarding utilizing ABA signals from multiple wheelsets.

Each handcraft feature has eight values derived from signals of the four axle boxes on each rail. The eight values are aggregated from all the eight available wheelsets by averaging them. This approach reduces uncertainties in SAWP of ABA signals since we found in our previous study that ABA signals from different axle boxes show slight differences, such as slight shifting of SAWP peak locations and slight differences in SAWP magnitudes, even though they correspond to the same rail [31]. Fig. 6 shows that different wheelsets exhibit slight variations in SAWP, for example, segment II-24. Table 1 shows the extracted features from different wheels and the EnVTQI while considering ABA signals from different wheelsets (see Fig. 9). The results indicate that utilizing ABA signals from multiple wheelsets can reduce variations in EnVTQI related to different wheel conditions and measurement uncertainty.

**Table 1:** Extracted features of segment II-24 from ABA signals from different wheels.

	Left Rail					Right Rail				
	w1	w2	w3	w4	avg	w1	w2	w3	w4	avg
***SD***_**S,*w*,*r***_	4.46 × 10^−7^	3.92 × 10^−7^	3.70 × 10^−7^	3.99 × 10^−7^	4.02 × 10^−7^	5.74 × 10^−7^	7.54 × 10^−7^	7.29 × 10^−7^	7.46 × 10^−7^	7.01 × 10^−7^
% diff from avg	11.02 %	−2.35 %	−7.99 %	−0.68 %		−18.03 %	7.54 %	4.04 %	6.45 %	
***AUC***_**S,*w*,*r***_	9.10 × 10^−5^	8.69 × 10^−5^	8.32 × 10^−5^	9.24 × 10^−5^	8.84 × 10^−5^	7.77 × 10^−5^	9.50 × 10^−5^	9.51 × 10^−5^	9.84 × 10^−5^	9.15 × 10^−5^
% diff from avg	2.97 %	−1.71 %	−5.79 %	4.53 %		−15.12 %	3.75 %	3.93 %	7.45 %	
***SD***_**B,*w*,*r***_	9.06 × 10^−7^	7.56 × 10^−7^	8.66 × 10^−7^	9.20 × 10^−7^	8.62 × 10^−7^	1.02 × 10^−6^	1.14 × 10^−6^	1.36 × 10^−6^	1.30 × 10^−6^	1.21 × 10^−6^
% diff from avg	5.13 %	−12.34 %	0.50 %	6.71 %		−15.36 %	−5.48 %	13.01 %	7.83 %	
***AUC***_**B,*w*,*r***_	4.28 × 10^−4^	4.14 × 10^−4^	3.77 × 10^−4^	4.39 × 10^−4^	4.14 × 10^−4^	4.00 × 10^−4^	4.57 × 10^−4^	5.42 × 10^−4^	5.06 × 10^−4^	4.76 × 10^−4^
% diff from avg	3.37 %	−0.18 %	−9.03 %	5.84 %		−16.02 %	−4.01 %	13.84 %	6.19 %

Then, five features are considered to determine the EnVTQI of a particular track segment. One feature is from the longitudinal level, *avg*(*SD*_LL_), and four features are from SAWP of ABA signals, *avg*(*SD*_S_), *avg*(*AUC*_S_), *avg*(*SD*_B_), and *avg*(*AUC*_B_).

EnVTQI of a particular track segment is a single value obtained from the fusion of the five features. However, directly fusing all features might cause bias since the units and ranges of features are different, as shown in the x-axis of Fig. 8. Therefore, the rescaling of each feature is applied. For a particular track segment, its corresponding features are rescaled with respect to the determined lower and upper values, as follows:

Eq. 8 $${\bar{F}}^{j}=\left(\frac{avg\left(F^{j}\right)-LV_{F}}{UV_{F}-LV_{F}}\right)$$

where ${\bar{F}}^{j}$ is a rescaled value of feature *F* at segment *j*, *avg*(*F* ^*j*^*)* is an averaged value of feature *F* at segment *j*, *LV*_*F*_ and *UV*_*F*_ are the determined lower and upper values of feature *F*. Those boundary values can be adjusted to the measured track. For this study, the lower and upper values were determined, as shown in Table 2, based on observation from Fig. 8.

**Table 2:** Lower and upper values per feature.

Features *F*	Description	Lower and upper values				Unit
		With speed influence reduction		Without speed influence reduction		
		Lower *LV*_*F*_	Upper *UV*_*F*_	Lower *LV*_*F*_	Upper *UV*_*F*_	
*avg*(*SD*_LL_)	the average of the standard deviation of *LL*_*r*_	3.0 × 10^−1^	3.0 × 10^0^	3.0 × 10^−1^	3.0 × 10^0^	mm
*avg*(*SD*_S_)	the average of the standard deviation of *SAWP*_S*,w,r*_	4.0 × 10^−8^	6.0 × 10^−6^	3.0 × 10^−3^	1.0 × 10^0^	m^2^/s^4^
*avg*(*AUC*_S_)	the average of the area under the curve of *SAWP*_S*,w,r*_	1.0 × 10^−5^	7.5 × 10^−4^	1.0 × 10^0^	1.2 × 10^2^	m^3^/s^4^
*avg*(*SD*_B_)	the average of the standard deviation of *SAWP*_B*,w,r*_	2.0 × 10^−7^	5.5 × 10^−5^	1.0 × 10^−2^	1.5 × 10^1^	m^2^/s^4^
*avg*(*AUC*_B_)	the average of the area under the curve of *SAWP*_B*,w,r*_	2.0 × 10^−4^	2.0 × 10^−3^	5.0 × 10^1^	5.8 × 10^2^	m^3^/s^4^

Then, the five rescaled features of segment *j*, ${\bar{SD}}_{\text{LL}}^{j}$, ${\bar{SD}}_{S}^{j}$, ${\bar{AUC}}_{S}^{j}$, ${\bar{SD}}_{B}^{j}$, and ${\bar{AUC}}_{B}^{j}$ are fused into *EnvTQI* ^*j*^*,* as follows:

Eq. 9 $$EnVTQI^{j}=\alpha_{1}{\bar{SD}}_{\text{LL}}^{j}+\alpha_{2}{\bar{SD}}_{S}^{j}+\alpha_{3}{\bar{AUC}}_{S}^{j}+\alpha_{4}{\bar{SD}}_{B}^{j}+\alpha_{5}{\bar{AUC}}_{B}^{j},\alpha_{1}+\alpha_{2}+\alpha_{3}+\alpha_{4}+\alpha_{5}=1$$

where *EnVTQI* ^*j*^ is the enhanced vertical track quality index of track segment *j*, and *α*_*i*_ is the weight factor in the range between 0.00 and 1.00 of parameter *i*, in which the sum of *α*_*i*_ for five features is 1. EnVTQI is unitless, where the segments with a lower EnVTQI value show a better track quality than those with a higher EnVTQI value. The procedure of tuning weight factors can be conducted following the reasoning behind the EN standard [5]. It also allows infrastructure managers to tune the importance of each handcraft feature. For instance, the weight of ballast-related features can be higher than substructure-related features in case the condition of the ballast layer requires more attention than the substructure. The weight of the substructure-related features can be set to 0.00 for some applications, such as planning for tamping.

## 4 Results and discussion

### 4.1 Vertical track quality analysis

#### 4.1.1 EN standard indices

EN 13848-6 [5] defines two approaches to quantify track quality. The first approach considers the overall track quality index, defined as the Combined standard deviation (CoSD), which can be calculated as follows.

Eq. 10 $$CoSD=\sqrt{\alpha_{\text{AL}}avg{\left(SD_{\text{AL}}\right)}^{2}+\alpha_{G}S{D_{G}}^{2}+\alpha_{\text{CL}}S{D_{\text{CL}}}^{2}+\alpha_{\text{LL}}avg{\left(SD_{\text{LL}}\right)}^{2}}$$

where *CoSD* is the combined standard deviation, *SD*_*i*_ is the standard deviation of the geometry parameter *i*, *α*_*i*_ is the weight factor of the geometry parameter *i*, AL is alignment, G is track gauge, CL is cross level, and LL is longitudinal level. The second approach considers a particular track geometry parameter. For vertical track quality, the average standard deviation of the longitudinal levels of the left and right rails, *avg*(*SD*_LL_), is considered.

**Fig. 10:**
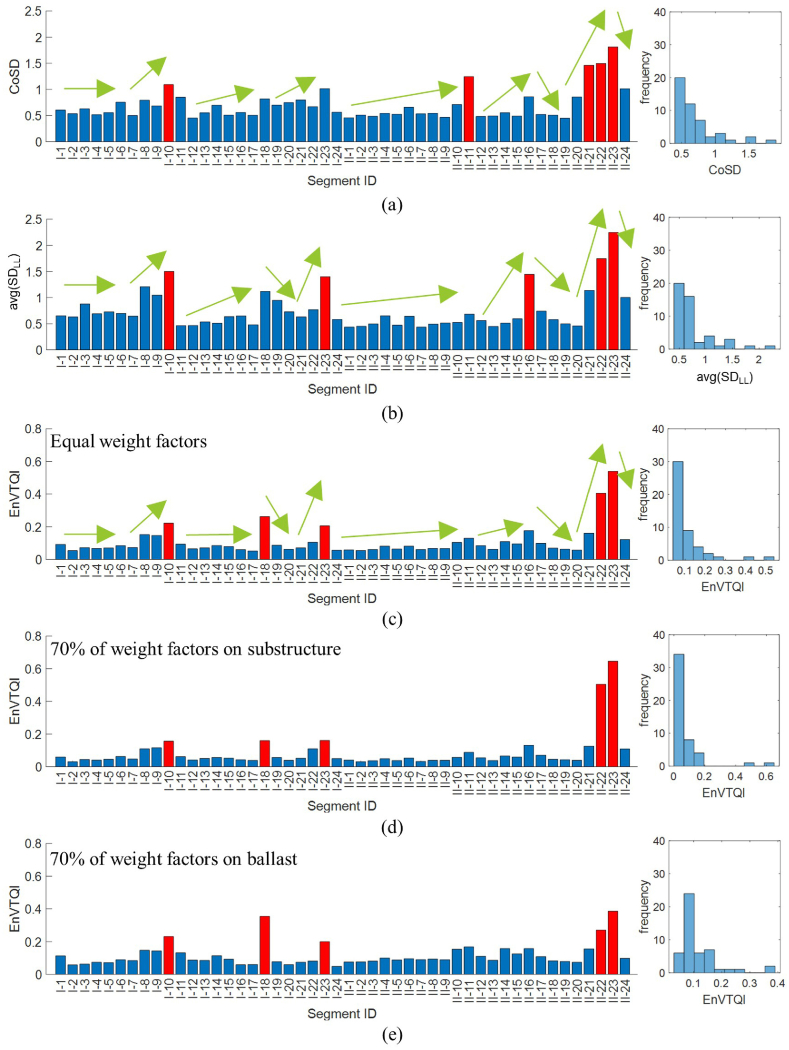
Track quality indices and its histogram of case study track segments based on measurement data in 2019: (a) CoSD where equal weight factors are assigned, (b) *avg*(*SD*_LL_), (c) EnVTQI where equal weight factors are assigned, (d) EnVTQI where 70 % of weight factors are assigned to substructure-related features, and (e) EnVTQI where 70 % of weight factors are assigned to ballast-related features. Red bars indicate track segments with the worst 10 % of all considered segments.

Track geometry data from a measurement in 2019 is selected to discuss the track quality of the case study track segments. Forty-eight track segments, namely I-1 to I-24 for Track-1 and II-1 to II-24 for Track-II, are selected as case studies. CoSD is calculated with an equal weighting factor of 0.25 for each track geometry parameter, as in Ref. [11], which is shown in Fig. 10(a), while *avg*(*SD*_LL_) is shown in Fig. 10(b).

Next, a comparison between CoSD and *avg*(*SD*_LL_) is conducted. The CoSD values of all the 48 track segments range from 0.447 to 1.814. The five segments with higher CoSD values (ranked highest to lowest) are segments II-23, II-22, II-21, II-11, and I-10. These segments are highlighted as red bars in Fig. 10(a). In comparison, the *avg*(*SD*_LL_) values of the segments range from 0.435 to 2.247. Segment II-23 has the poorest vertical track condition with the highest *avg*(*SD*_LL_) value. The following four track segments are II-22, I-10, II-16, and I-23, in descending order of severity. In addition, the changing patterns of track quality evaluated by CoSD and *avg*(*SD*_LL_) are almost similar, as indicated by the arrows in Fig. 10(a and b). The Pearson correlation between CoSD and *avg*(*SD*_LL_) is 0.81. Furthermore, a similar distribution of their corresponding histograms of both indices can be observed. These findings align with the finding from Ref. [25] that the vertical track quality highly dominates the overall track quality. Hence, this study mainly focuses on the vertical track quality to represent overall track quality.

#### 4.1.2 Comparison between avg(SD_LL_) and EnVTQI

The vertical track quality evaluated by EnVTQI for each case study track segment, calculated with an equal weight of 0.2 for each feature, is shown in Fig. 10(c). We observe that *avg*(*SD*_LL_) and EnVTQI indicate some segments have similar patterns. For instance, in Track-II, the track quality increases from II-16 to II-20, then the track quality drops from II-21 to II-23, and the track quality increases again in II-24. The Pearson correlation between *avg*(*SD*_LL_) and EnVTQI is 0.91. Moreover, the histogram of EnVTQI shows good similarity to the histogram of *avg*(*SD*_LL_).

**Fig. 11:**
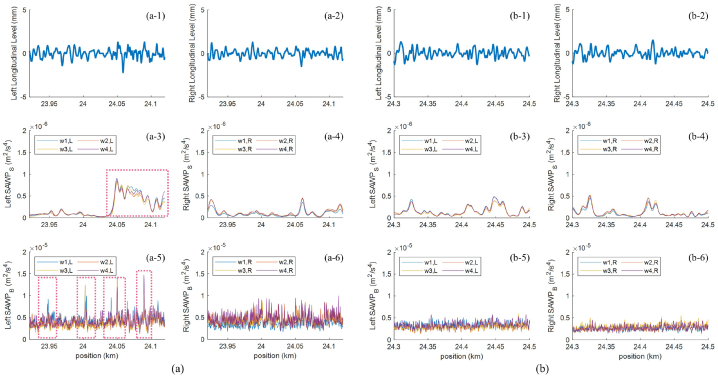
Signals corresponding to the track segment: (a) track segment I-11, (b) track segment I-12. Subfigures 1 and 2 are longitudinal levels at the left and right rails, subfigures 3 and 4 are *SAWP*_S_ at the left and right rails, and subfigures 5 and 6 are *SAWP*_B_ at the left and right rails.

There are segments of interest when comparing *avg*(*SD*_LL_) and EnVTQI, such as Segments I-11 and I-12. From the perspective of longitudinal levels, the vertical track quality of segments I-11 and I-12 can be considered similar since the *avg*(*SD*_LL_) values of I-11 and I-12 are 0.464 mm and 0.467 mm, respectively. However, based on the EnVTQI with equally assigned weighting factors in Fig. 10(c), the track condition of segment I-11 is more severe than segment I-12 since EnVTQI ^I−11^ (0.093) is higher than EnVTQI ^I−12^ (0.064). While considering features for determining EnVTQI, as shown in Fig. 11(a-3 – a-6, b-3 – b-6), all ABA-derived features corresponding to segment I-11 provide higher values than segment I-12, as shown in Table 3. Considering Figs. 11(a-1, a-2, b-1, b-2), no noticeable changes in longitudinal level signals can be found in segments I-11 and I-12. In comparison, changes in SAWP_S_ and SAWP_B_ at track positions indicated by the dashed boxes in Fig. 11(a-3and 11a-5) can be noticed, which leads to the higher ABA-derived features of segment I-11. These findings suggest that using ABA signals to assess track condition can enhance the standard practice of only considering track geometry.

**Table 3:** Parameters corresponding to considered track segments.

Parameter	Description	I-11	I-12	Relative difference (%)	Ratio I-11: I-12	I-18	II-16	Relative difference (%)	Ratio I-18: II-16
$avg\left(SD_{\text{LL}}\right)$	the average of the standard deviation of *LL*_*r*_	0.464	0.467	0.6	0.99	1.119	1.446	29.2	0.77
${\bar{SD}}_{\text{LL}}$	the rescaled of *avg*(*SD*_LL_)	0.061	0.062	1.6	0.98	0.303	0.425	40.3	0.71
${\bar{SD}}_{S}$	the rescaled of *avg*(*SD*_S_)	0.023	0.010	56.5	2.30	0.035	0.070	100.0	0.50
${\bar{AUC}}_{S}$	the rescaled of *avg*(*AUC*_S_)	0.038	0.027	28.9	1.41	0.081	0.106	30.9	0.76
${\bar{SD}}_{B}$	the rescaled of *avg*(*SD*_B_)	0.017	0.006	64.7	2.83	0.431	0.018	95.8	23.94
${\bar{AUC}}_{B}$	the rescaled of *avg*(*AUC*_B_)	0.326	0.216	33.7	1.51	0.463	0.263	43.2	1.76
EnVTQI	the enhanced vertical track quality index	0.093	0.064	31.2	1.45	0.263	0.176	33.1	1.49

**Fig. 12:**
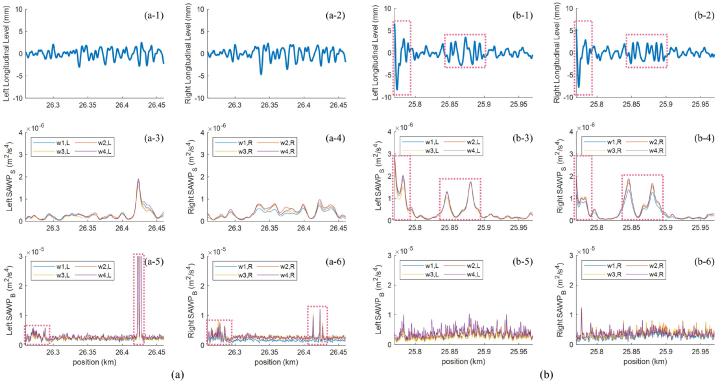
Signals corresponding to the track segment: (a) track segment I-18, (b) track segment II-16. Subfigures 1 and 2 are longitudinal levels at the left and right rails, subfigures 3 and 4 are *SAWP*_S_ at the left and right rails, and subfigures 5 and 6 are *SAWP*_B_ at the left and right rails.

Other locations of interest are I-18 and II-16. EnVTQI indicates that segment I-18 is one of the worst segments, instead of segment II-16 as indicated by *avg*(*SD*_LL_). Considering longitudinal levels, as shown in Fig. 12(a-1, a-2, b-1, b-2), it can be noticed from the dash boxes in Figs. 12(b-1 and b-2) that segment II-16 provides a higher variation of longitudinal levels than segment I-18. The values of *avg*(*SD*_LL_) for II-16 and I-18 are 1.446 mm and 1.118 mm, respectively. In the case of EnVTQI, the ${\bar{SD}}_{S}$ and ${\bar{AUC}}_{S}$ corresponding to segment II-16 are higher than those of segment I-18, as shown in Table 3. While considering the remaining features, as shown in Figs. 12(a-3 – a-6, b-3 – b-6), the dashed boxes in Figs. 12(b-3 and b-4) indicate the locations with noticeable *SAWP*_S_ peaks, which lead to the high values of the ${\bar{SD}}_{S}$ and ${\bar{AUC}}_{S}$. Thus, according to these features, the worst substructure condition is from segment II-16. However, features related to the ballast layer condition, ${\bar{SD}}_{B}$ and ${\bar{AUC}}_{B}$, of segment II-18 are significantly higher than those of segment II-16, as shown in Table 3. In Figs. 12(a-5 and a-6), dash boxes indicate the locations that cause the high value of these features. The most noticeable peaks are located approximately at km 26.42, indicating the most severe spot in the ballast layer of segment II-18. This spot significantly dominates the overall track condition, leading to a higher value of *EnVTQI* ^I−18^ (0.263) than *EnVTQI* ^II−16^ (0.176). Regarding relative percentage differences, we observe more considerable variations for I-18 regarding the ballast features, so it is reasonable to suggest that the location faces a higher degradation condition. Finally, while EnVTQI provides aggregated information about track quality, the values required to calculate EnVTQI allow for identifying problematic track structure layers, allowing the physical interpretability of the results.

### 4.2 EnVTQI under speed variation

**Fig. 13:**
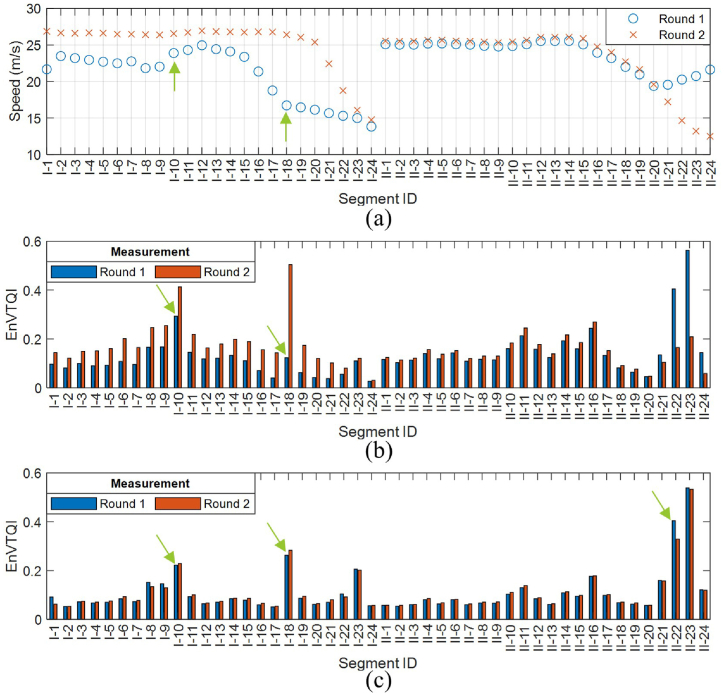
Comparison of EnVTQI between 2 rounds of ABA measurement: (a) measurement speeds from 2 rounds at a particular segment, (b) EnVTQI from ABA signals without reducing the influence of speed variation, (c) EnVTQI from ABA signals with reducing the influence of speed variation.

ABA signals are speed-dependent, and in operational conditions, it is not possible to conduct ABA measurements with a constant speed for the whole railway line. In this subsection, the performance of the method to reduce the effect on speed is investigated. The average speed of the instrumented wagon passing by each track segment is calculated. The speed profiles from 2 measurement rounds along the case study railway line are shown in Fig. 13(a). It can be noticed that the measurement speeds from the two rounds are different, especially from section I-1 to section I-22 and from section II-21 to section II-24. The lowest speed discrepancy is 0.2 m/s at segment II-20, while the highest speed variation is 9.7 m/s at segment I-18.

The EnVTQIs calculated from the ABA signals without and with speed influence reduction are shown in Fig. 13(b and c), respectively. Without speed correction, considering segments I-10 and I-18 indicated by arrows in Fig. 13(b), EnVTQI corresponding to measurement round 1 suggests that the condition of segment I-10 is more severe than that of segment I-18, while EnVTQI corresponding to measurement round 2 suggests the opposite result. The reason for this is that the speed of segment I-10 (23.9 m/s) is higher than that of segment I-18 (16.7 m/s) in measurement round 1, while the speeds of both segments can be considered similar (26.6 m/s for segment I-10 and 26.4 m/s for segment I-18) in measurement round 2. In measurement round 2, where speed differences can be neglected, it can be concluded that the condition of segment I-18 is more severe than that of segment I-10. This is in line with the results evaluated by the EnVTQI with speed correction (Fig. 13(c)). This simple example demonstrates the need and the capability of the proposed approach to reduce the influence of speed variation.

The total differences of EnVTQIs from the two ABA measurement rounds are 45.8 % if the proposed approach is not utilized. In contrast, the total differences are reduced to 6.6 % after utilizing the proposed approach. Significant EnVTQI differences still exist in some track segments, for example, segment II-22, indicated by an arrow in Fig. 13(c).

### 4.3 Influence of weight factors on EnVTQI

Different assigned weight factors allow users to tune the importance of features that are $\alpha_{1}$ for ${\bar{SD}}_{\text{LL}}$ related to longitudinal levels, $\alpha_{2}$ for ${\bar{SD}}_{S}$ and $\alpha_{3}$ for ${\bar{AUC}}_{S}$ related to the condition of the substructure layer, $\alpha_{4}$ for ${\bar{SD}}_{B}$ and $\alpha_{5}$ for ${\bar{AUC}}_{B}$ related to the condition of the ballast layer. Tuning weight factors makes EnVTQI better reflect track condition for a particular application, such as tamping, and better match the characteristics of a particular railway line. In this section, characteristics of EnVTQI regarding various assigned weight factors are investigated.

**Fig. 14:**
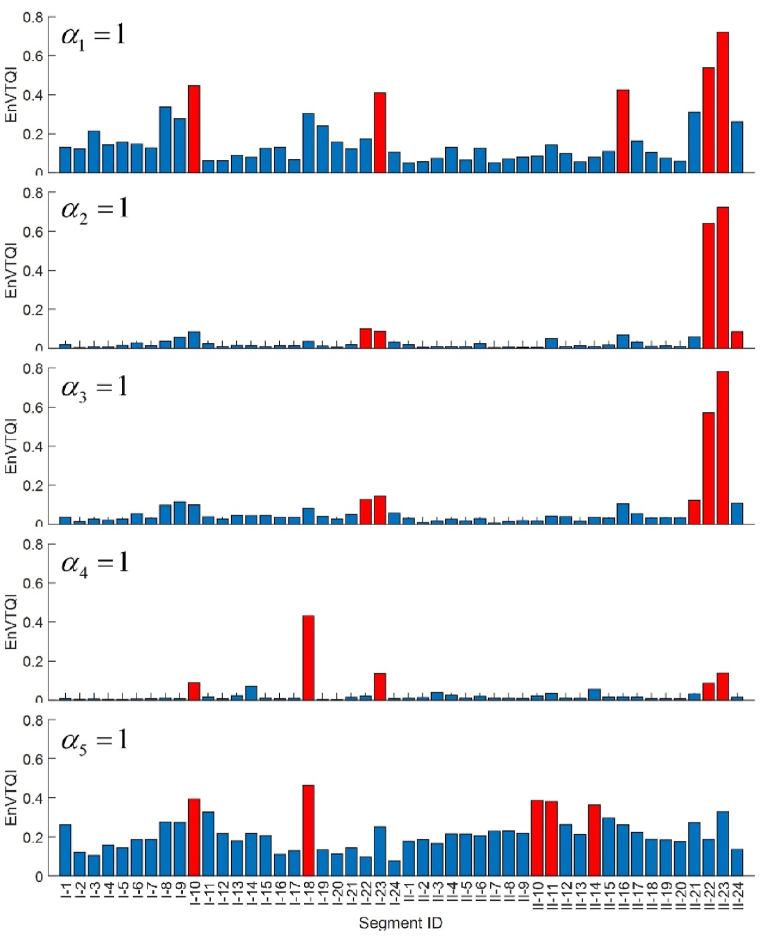
EnVTQI of the case study track segments in 2019 regarding different weight factors. Red bars indicate track segments with the worst 10 % of all considered segments.

Fig. 14 shows the EnVTQI of 48 case study segments based on various combinations of weight factors, in which one weight is set to 1.00, and the remaining weights are set to 0.00. The results demonstrate that different weight factor combinations yield different EnVTQI characteristics, indicating the differences in sensitivity of specific features and ballast and substructure conditions.

Three combinations of weight factors are tested, and their corresponding EnVTQI are shown in Fig. 10. Firstly, equal weight factors of 0.20 are assigned to all features, and EnVTQI can be found in Fig. 10(c). Secondly, 70 % of the total weight is assigned to substructure-related features. The weight factors of rescaled ${\bar{SD}}_{S}$ and ${\bar{AUC}}_{S}$ are 0.35 each, while the weight factors of the remaining features are 0.10 each. The corresponding EnVTQI to this configuration can be found in Fig. 10(d). Lastly, 70 % of the total weight is assigned to ballast-related features. Weight factors of 0.35 are assigned to rescaled ${\bar{SD}}_{B}$ and ${\bar{AUC}}_{B}$, while the remaining features are assigned a weight factor of 0.10. The results of EnVTQI corresponding to this weight configuration are shown in Fig. 10(e).

By determining the 10 % of track segments with the highest EnVTQI, the same five segments, II-23, II-22, I-18, I-10, and I-23, are indicated by EnVTQI from three different weight configurations. Differences in the severity ranking and the magnitude of EnVTQI can be observed and shown in Table 4. In the case of high-weight factors assigned to substructure-related features, the severity of segments II-23 and II-22 are more distinguishable. In the case of high-weight factors assigned to ballast-related features, segment I-18 is in the second rank, and its EnVTQI magnitude is close to segment II-23, the most severed segment. This finding agrees with the finding in Section 4.1.2 that a high *SAWP*_B_ peak can be found at segment I-18.

**Table 4:** The 10 % track segments with the highest EnVTQI.

Severity rank 1 to 5 from higher to lower severity	Segment and its EnVTQI					
	Equal weight		70 % of the weight on substructure features		70 % of the weight on ballast features	
1	II-23	0.539	II-23	0.646	II-23	0.386
2	II-22	0.405	II-22	0.505	I-18	0.355
3	I-18	0.263	I-23	0.161	II-22	0.271
4	I-10	0.222	I-18	0.160	I-10	0.231
5	I-23	0.206	I-10	0.157	I-23	0.200

### 4.4 Evolution of EnVTQI over time

**Fig. 15:**
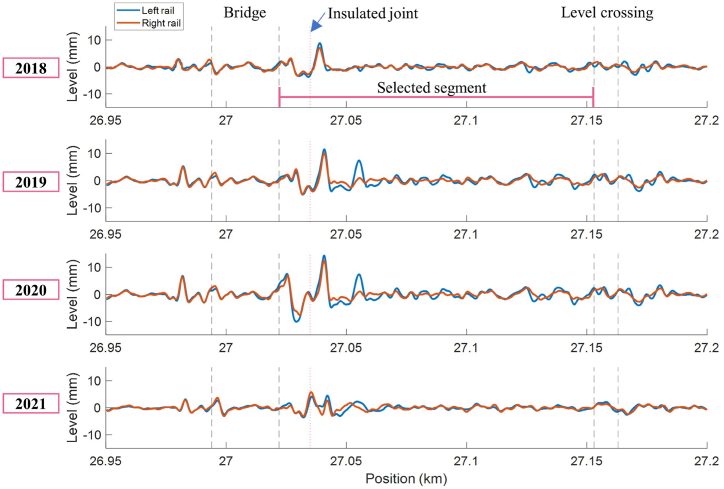
Evolution of longitudinal levels from 2018 to 2021.

**Fig. 16:**
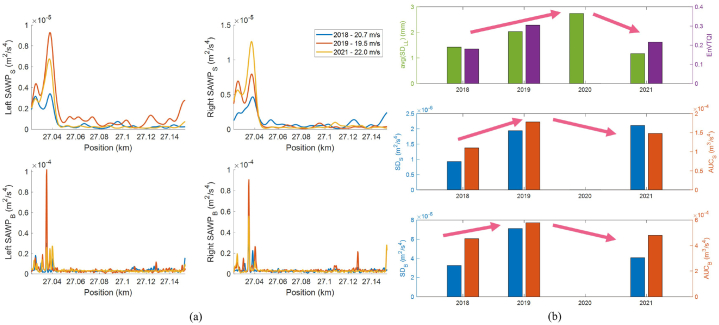
(a) *SAWP*_S_ and *SAWP*_B_ at the left and right rails from 3 different measurement years, (b) the evolution of EnVTQI and the corresponding features over time.

Fig. 15 shows historical longitudinal levels on a track that suggests a degradation pattern near the bridge from 2018 to 2021. The track quality in terms of *avg*(*SD*_LL_), shown in Fig. 16(b), continuously increased from 1.423 in 2018 to 2.742 in 2020 and then significantly dropped to 1.168 in 2021, meaning an improvement in track quality. This finding suggests that the major track maintenance activities were conducted between 2020 and 2021.

Considering features from the available ABA signals measured in 2018, 2019, and 2021, drastic changes in SAWP can be observed near the bridge, as shown in Fig. 16(a). Extracted features from *SAWP*_S_ and *SAWP*_B_ indicate the same trend as the longitudinal levels, where the feature values increase between 2018 and 2019 and later decrease in 2021, as shown in Fig. 16(b). These changes over time resulted in the evolution of EnVTQI following a similar pattern. The EvTQI with equal weight factors for all inputs increased by 67.4 % in 2019, showing worse track conditions than in 2018, and decreased by 57.4 % in 2021, indicating better track conditions than in 2019.

**Fig. 17:**
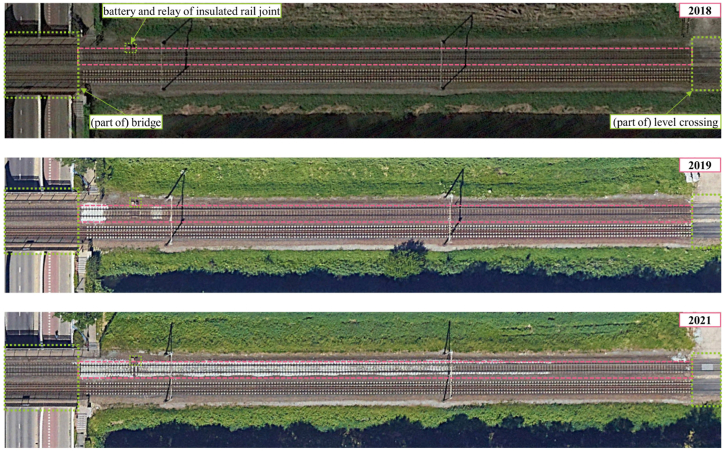
Historical satellite images of a considered segment (source of satellite images: Google Earth).

To support the abovementioned statement, the historical satellite images from 2018, 2019, and 2021, as shown in Fig. 17, suggest evidence of track maintenance activities. Even though the exact types of track maintenance cannot be indicated, the changes in ballast appearance over time are due to new ballast placement, according to our track investigation in 2021. Differences in the satellite images between 2018 and 2019 indicate minor maintenance activities, especially near the bridge. Additionally, relatively major activities were conducted in 2021 since the widespread difference in ballast appearance between 2019 and 2021 can be observed, resulting in a significant improvement in track quality in 2021.

## 5 Conclusions and suggestions for further works

A framework for designing an enhanced vertical track quality index, EnVTQI, is proposed. The proposed framework integrates features derived from longitudinal levels and ABA signals, in which two wavelengths of ABA signals are considered to identify track conditions at the substructure and ballast layers. In addition, this framework can be applied to railway networks with various measurement conditions, such as measurement speeds, because of the proposed approach to reducing the influence of speed variation. The performance of EnVTQI is evaluated using measured datasets of the Dutch railway line by comparing EnVTQI with *avg*(*SD*_LL_), an EN standard vertical TQI. The track condition of the study line is generally good. EnVTQI captures local changes in the condition of substructure and ballast layers, making it possible to perform better in indicating segments with poor track condition, including the effect of both deviations in track geometry and dynamic responses. The proposed method to reduce the influence of variation in measurement speeds on ABA features is based on an empirical approach and allows the use of the EnVTQI considering train in normal operations.

As part of future research, CoSD, the overall track quality index according to EN 13848-6, was developed based on longitudinal level, alignment, gauge, and cross level. Those track geometry parameters correspond to the condition of railway tracks in both vertical and lateral directions. Therefore, an enhanced TQI derived from vertical, lateral, and longitudinal train-track interaction could be considered. This is important for the analysis of curves. In addition, long-term periodic change trend analysis with further validation campaigns in various locations under various measurement conditions is to be considered. Finally, efforts towards standardizing this method for the use of vehicle responses to improve TQIs are required.

## Funding

This research was partly supported by 10.13039/501100009957ProRail and Europe's Rail Flagship Project IAM4RAIL - Holistic and Integrated Asset Management for Europe's RAIL System. Funded by the 10.13039/501100000780European Union. Views and opinion expressed are however those of the authors(s) only and do not necessarily reflect those of the European Union. Neither the 10.13039/501100000780European Union nor the granting authority can be held responsible for them. This project has received funding from the European Union's 10.13039/100018693Horizon Europe research and innovation programme under Grant Agreement No 101101966. The first and second authors would like to thank the Royal Thai Government for their Ph.D. scholarship.

## Ethics and consent statement

Review and approval by an ethics committee were not needed because this work did not involve any human or animal subjects.

## Data availability statement

The data associated with this work has not been deposited into a publicly available repository. The data will be made available on request.

## CRediT authorship contribution statement

**Siwarak Unsiwilai:** Writing – review & editing, Writing – original draft, Visualization, Validation, Software, Methodology, Investigation, Formal analysis, Data curation, Conceptualization. **Wassamon Phusakulkajorn:** Writing – review & editing, Validation, Software, Investigation, Formal analysis. **Chen Shen:** Writing – review & editing, Visualization, Validation, Investigation, Formal analysis, Conceptualization. **Arjen Zoeteman:** Writing – review & editing, Validation, Resources, Project administration, Funding acquisition, Conceptualization. **Rolf Dollevoet:** Writing – review & editing, Resources, Project administration, Funding acquisition, Conceptualization. **Alfredo Núñez:** Writing – review & editing, Writing – original draft, Visualization, Validation, Supervision, Resources, Project administration, Methodology, Investigation, Funding acquisition, Formal analysis, Conceptualization. **Zili Li:** Writing – review & editing, Supervision, Resources, Project administration, Funding acquisition, Formal analysis, Conceptualization.

## Declaration of competing interest

The authors declare that they have no known competing financial interests or personal relationships that could have appeared to influence the work reported in this paper.
